# Missed opportunities in aspirin prescribing for preeclampsia prevention

**DOI:** 10.1186/s12884-023-06039-w

**Published:** 2023-10-07

**Authors:** Noreen Singh, Saskia Shuman, Jacqueline Chiofalo, Mariela Cabrera, Aimee Smith

**Affiliations:** 1 The Institute for Family Health, 230 W 17th St, New York, NY 10011 USA; 2 Mount Sinai Family Medicine Residency Program, New York, NY USA; 3 Icahn School of Medicine at Mount Sinai, New York, NY USA; 4 Boston University, Boston, MA USA; 5 The Institute for Family Health, Kingston, NY USA; 6 Mid-Hudson Family Medicine Residency Program, Kingston, NY USA

**Keywords:** Preeclampsia, Aspirin, Hypertension, Maternal mortality, FQHC, Maternal morbidity

## Abstract

**Background:**

Hypertensive disorders of pregnancy, including preeclampsia, are a leading cause of perinatal morbidity and mortality in the United States, particularly among low-income and historically marginalized populations. Evidence suggests low-dose aspirin prophylaxis may help prevent preeclampsia in individuals at increased risk of developing the disease. This study examines associations between preeclampsia risk factors and aspirin prescribing practices among patients receiving prenatal care at a network of federally qualified health centers (FQHC).

**Methods:**

Researchers conducted retrospective chart reviews (n = 523) of pregnant individuals ages 18–50 who completed two or more prenatal visits at the FQHC between January 1, 2019 and December 31, 2020. Prescription patterns for patients at moderate and high risk for preeclampsia were analyzed using unadjusted and adjusted logistic regression models to identify the patients with the greatest risk of not receiving the recommended prophylactic treatment.

**Results:**

Of 249 total patients considered at risk for preeclampsia, only 39% received an aspirin prescription. 57.89% of patients with any high-risk factor were appropriately prescribed aspirin, but only 27.27% of patients with two or more moderate-risk factors without high-risk factors received a prescription. Clinicians most frequently prescribed aspirin for patients with a history of preeclampsia and history of hypertension. However, aspirin was prescribed a maximum of 78.79% of the time for patients with a prior history of hypertension. Among moderate-risk factors, patients with advanced maternal age, Black race, or nulliparity were significantly more likely in adjusted models to be prescribed aspirin.

**Conclusions:**

Despite the documented benefits of aspirin prescribing and support from professional societies, there are still many missed opportunities for aspirin prophylaxis to prevent preeclampsia. Future interventions should focus on identifying patients who qualify for aspirin prophylaxis on the basis of having multiple moderate-risk factors without comorbid high-risk factors.

## Background

Hypertensive disorders of pregnancy (HDP), such as preeclampsia, are a leading cause of perinatal morbidity and mortality in the United States; the majority of these complications and deaths are thought to be preventable [1]. The maternal mortality rate in the United States is higher than all other wealthy countries, despite disproportionately higher health care expenditure [2]. Moreover, significant racial disparities persist in maternal mortality (MM) and severe maternal morbidity (SMM), with Black pregnant individuals experiencing 2–4 times higher pregnancy-related death and complication rates than their White counterparts [3]. New York State has historically had higher SMM rates than the national average, and this rate has been increasing over the last decade [4]. As part of the effort to reduce MM and SMM moving forward, detailed maternal health data collection is necessary, including social determinants of health.

Global rates of preeclampsia (PEC) range from 2 to 8% of pregnancies [5]. There is a growing body of literature demonstrating the increased prevalence of hypertensive disorders of pregnancy among low-income and historically marginalized populations, which is consistent with the patient population served at federally qualified health centers (FQHCs) [6, 7]. Most prominent in the literature are the racial and ethnic disparities in HDP prevalence and related adverse outcomes, which persist even after adjusting for socioeconomic factors. Previous research has established that people with higher socioeconomic status generally experience lower cardiovascular risk and better health outcomes compared to those in lower socioeconomic groups [8]. However, recent studies have suggested that in the United States, higher socioeconomic status may not confer the same health benefits in attenuating preeclampsia risk to Black pregnant individuals compared to White pregnant individuals [9]. There are few studies in the United States examining interventions for preeclampsia prevention among a patient population where a majority are affected by these socioeconomic and racial and ethnic disparities.

Low-dose aspirin is currently the only intervention recommended by the United States Preventive Services Task Force (USPSTF) and American College of Obstetricians (ACOG) for primary prevention of preeclampsia in individuals at increased risk of developing the disease [10]. In systematic reviews, aspirin demonstrated a preeclampsia risk reduction up to 25%, leading the USPSTF to publish guidelines in 2014 recommending aspirin for pregnant individuals with any high-risk factors and considered for those with two or more moderate-risk factors [11]. Updated guidelines from USPSTF and ACOG in December 2021 continue to recommend aspirin for individuals with more than one preeclampsia moderate-risk factor [12]. The update further clarifies that “Black persons” and “lower income” were included as moderate-risk factors due to social, rather than biological, factors that increased risk due to inequitable care [13].

The goal of this study is to examine the associations between preeclampsia risk factors and aspirin prescribing practices among patients receiving prenatal care at one of the largest federally qualified health center (FQHC) networks in New York State. By analyzing the prescribing patterns for patients at moderate and high risk for preeclampsia, we aim to identify which patients remain at highest risk of not receiving the recommended prophylactic treatment despite ACOG guidelines. Addressing disparities in aspirin prescribing in FQHC settings where the majority of patients are individuals of color, low-income, and/or publicly insured or uninsured has important implications in addressing the persistent racial and income-based disparities in maternal health care.

## Methods

### Patient selection

This is a retrospective cohort study utilizing chart review of electronic health records, with specific inclusion criteria including pregnant individuals ages 18–50 who received two or more prenatal visits at any Institute for Family Health site in New York State between January 1, 2019 and December 31, 2020. Pregnant individuals under 18 years of age, patients who had not given birth between 2019 and 2021, and patients with pregnancy losses prior to 22 weeks were excluded.

### Data collection

We extracted a list of patients with an episode of pregnancy between calendar year 2019 and 2020. Patient charts were reviewed initially at random, then patients with ACOG risk factors were identified and received chart reviews. Maternal, obstetric, and sociodemographic data were obtained from the electronic medical record, both at Institute for Family Health as well as delivery hospitals, if available. Patient medical records were examined for aspirin prescribing during the course of the pregnancy episode. All doses used in this study were 81 mg daily. Study data were collected and managed using REDCap electronic data capture tool hosted at the Institute for Family Health [14, 15].

Variables examined include ACOG risk factors: age and parity at time of delivery, race, granular ethnicity (self-reported by patients), private vs. public insurance, pre-pregnancy BMI, prior history of preeclampsia, chronic hypertension, diabetes mellitus, chronic kidney disease, autoimmune disease, preterm birth, low birth weight/small for gestational age infant, current multifetal gestation, interpregnancy interval greater than 10 years, and family history of preeclampsia. Outcomes of interest were aspirin initiation during pregnancy of interest and gestational age at initiation. If there was documentation of a historical aspirin prescription in the patient’s electronic medical record during the pregnancy, the patient was deemed to have been prescribed low-dose aspirin.

Preeclampsia risk was assigned based on existing ACOG definitions; patients were considered at high risk if they had a history of preeclampsia in a previous pregnancy, diabetes, chronic hypertension, multifetal gestation, autoimmune disease such as antiphospholipid syndrome, or chronic kidney disease. Patients were considered at moderate risk for preeclampsia if they had nulliparity, obesity (pre-pregnancy BMI > 30), Black race, a family history of preeclampsia in mother or sister, were advanced maternal age (AMA), defined by age 35 years or older by the time of birth, previous low birth weight or small for gestational age infant, previous adverse pregnancy outcome, or greater than 10 year interpregnancy interval [13]. Patients were eligible for aspirin prescribing if they had one or more high-risk factor, or if they had two or more moderate-risk factors for preeclampsia. The primary outcome of interest was the rate of appropriate aspirin prescribing in patients with high-risk factors and combinations of moderate and high-risk factors for preeclampsia.

### Data analysis

Data was exported and analyses were performed using STATA version 17.0 (StataCorp LLC, College Station, TX). Data on aspirin prescribing during the course of the pregnancy episode was coded as dichotomous variables; missing values for aspirin prescribing were re-coded as no aspirin prescribed. Moderate and high-risk factors as defined by the American College of Obstetricians and Gynecologists (ACOG) and United States Preventive Services Task Force (USPSTF) were identified and recoded to signify presence or absence of the risk factor; variables with missing values were recoded as absence of the risk factor.

We conducted unadjusted and adjusted logistic regression models to examine each risk factor in relation to prescription of aspirin throughout the course of the pregnancy episode. Three additional models were created to look at the odds of aspirin prescribing based on an aggregate of risk factors: any factor determined as high risk by ACOG, two or more factors determined as moderate risk by ACOG, and presence of any ACOG risk factor regardless of its classification as high or moderate risk.

## Results

The research team reviewed and included a total of 523 charts in the analysis. Table 1 presents demographic information about the patients. The largest race/ethnicity group in our sample was Hispanic (40.2%), followed by Black/African American (27.9%), non-Hispanic White (25%), and Other (6.9%). Our sample was relatively evenly distributed across age groups, with 23.5% aged 18–24, 56% aged 25–34, and 20.5% aged 35 or older. The majority of individuals utilized Medicaid insurance (80.3%). During the study period, 78.78% of individuals did not receive aspirin, while 21.22% were prescribed low-dose aspirin.

**Table 1:** Demographics

**Race/Ethnicity**	N	%
Non-Hispanic White	131	25.0
Non-Hispanic Black/African American	146	27.9
Hispanic	210	40.2
Other	36	6.9
**Age Groups**		
18–24	123	23.5
25–34	293	56.0
35+	107	20.5
**Current Insurance Status**		
Commercial	99	18.93
Medicare	2	0.38
Medicaid	420	80.3
Dual Medicare Medicaid	2	0.38
**Aspirin**		
No ASA	412	78.78
ASA	111	21.22

Abbreviations: ASA, Aspirin

Table 2 demonstrates aspirin prescribing by risk category. Of the 523 total patients in this study, 47.6% were classified as being at increased risk for developing preeclampsia and eligible for low-dose aspirin per ACOG guidelines. Among the 249 patients who met the treatment guidelines, at least one high-risk factor was present in 18.16% and two or more moderate-risk factors (but no high-risk) were present in 35.98%. Among the patients with at least one high-risk factor present, 57.89% received aspirin. Among the 154 patients who had two or more moderate-risk factors but no high-risk factors, only 27.27% were prescribed aspirin. Patients with a high-risk factor were 3.66 times (CI 2.13, 6.29; p < 0.001) more likely to be prescribed aspirin compared to those with just moderate-risk factors.

**Table 2:** Aspirin prescribing by risk category

Risk Factor	n (N = 523)	% No ASA Rx	% ASA Rx	Unadj. OR	95% CI	p value Tar
Any High Risk	95	42.11	57.89	3.66*	2.13, 6.29	< 0.001
Two or more Moderate-Risk Factors, no high-risk factors	154	72.73	27.27			
Total combined risk pool appropriate for prescribing	249	61.04	38.96	11.85^	6.53, 21.48	< 0.001

Abbreviations: ASA, Aspirin; RF, Risk Factors

*compared to patients with two or more moderate-risk factors

^compared to patients without any risk factors

Table 3 presents preeclampsia risk factors and their association with low-dose aspirin prescribing during pregnancy, unadjusted and adjusted for other risk factors. Within the unadjusted model, four moderate-risk factors were significantly associated with aspirin prescribing, including AMA (OR 2.05, CI 1.27–3.301, p = 0.003), Black race (OR 2.63, CI 1.71–4.04, p < 0.001), family history PEC (OR 7.66, CI 1.38-42.399, p = 0.02), and nulliparity (OR 2.3, CI 1.33–3.99, p = 0.003). Significant high-risk factors within the unadjusted model were history of preeclampsia (OR 14.56, CI 6.6–32.1, p < 0.001), chronic hypertension (OR 17.908, CI 7.52–42.6, p < 0.001), and history of diabetes (OR 3.24, CI 1.31–8.05, p = 0.011). In the adjusted model, AMA (aOR 2.79, CI 1.51–5.17, p = 0.001), Black race (aOR 2.52, CI 1.48–4.28, p = 0.001), nulliparity (aOR 2.9, CI 1.46–5.76, p = 0.002), history of preeclampsia (aOR 20.81, CI 8.51–50.87, p < 0.001), and chronic hypertension (aOR 13.57, CI 5.05–36.49, p < 0.001) remained significantly associated with aspirin prescribing.

**Table 3:** Aspirin prescribing by preeclampsia risk factors

					ASA* at any time during pregnancy unadjusted for other RF N = 523			ASA at any time during pregnancy adjusted for other RF N = 523		
	Risk Factor	n	% No ASA Rx	% ASA Rx	unadj OR	95% CI	p value	adj OR	95% CI	p value
High Risk	History Preeclampsia	36	25	75	14.56	6.6, 32.11	< 0.001	20.81	8.51, 50.87	< 0.001
	Chronic Hypertension	33	21.21	78.79	17.908	7.52, 42.61	< 0.001	13.57	5.05, 36.49	< 0.001
	Pregestational Diabetes	20	55	45	3.24	1.31, 8.05	0.011	1.69	0.51, 5.54	0.385
	Chronic Kidney Disease	2	100	0	omit			omit		
	Autoimmune Disease	14	78.57	21.43	1.02	0.28, 3.72	0.974	0.97	0.186, 5.04	0.972
	Multifetal gestation	7	71.43	28.57	1.52	0.291, 7.95	0.619	3.18	0.534, 18.99	0.204
Mod. Risk	Advanced Maternal Age	107	68.22	31.78	2.05	1.27, 3.301	0.003	2.79	1.51, 5.17	0.001
	Black race	167	67.07	32.93	2.63	1.71, 4.04	< 0.001	2.52	1.48, 4.28	0.001
	Obesity	257	76.26	23.74	1.344	0.882, 2.04	0.168	1.1	0.663, 1.84	0.701
	Family history Preeclampsia	6	33.33	66.67	7.66	1.38, 42.399	0.02	5.49	0.797, 37.81	0.084
	> 10 year pregnancy interval	22	68.18	31.82	1.78	0.707,4.48	0.22	2.73	0.983, 7.60	0.054
	Nulliparity	68	64.71	35.29	2.307	1.33, 3.99	0.003	2.9	1.46, 5.76	0.002

Abbreviations: ASA, Aspirin; RF, Risk Factors

Table 4 shows a cross tabulation of the percentage of patients with more than one risk factor per ACOG guidelines who received aspirin through the clinical course of their pregnancy episode. Only risk factors with statistically significant odds associated with aspirin prescribing in the logistic models were included in the cross-tabulation.

Amongst high-risk factors, the data show that 75% of patients with a history of preeclampsia were prescribed aspirin, whether they had any additional risk factors or not. Patients with both a history of preeclampsia and chronic hypertension received aspirin 87.5% of the time. Similarly, 45% of patients with diabetes were prescribed aspirin, but when combined with chronic hypertension, 100% of eligible patients received aspirin.

Amongst moderate-risk factor combinations, AMA and obesity (27.12%), Black race and obesity (32.14%), and Black race and AMA (48.39%) were the risk profiles that had the lowest rates of appropriately prescribed aspirin for eligible patients. When these individual moderate-risk factors were combined with a high-risk factor, the proportion of patients prescribed aspirin increased.

**Table 4:** Proportion of aspirin prescribing by risk factor combination

Risk Factor	High Risk Factors			Moderate Risk Factors		
**% Aspirin Prescribing Based on Combination of Risk Factors**	**History Preeclampsia**	**Chronic Hypertension**	**Pre-gestational Diabetes**	**Advanced Maternal Age**	**Black race**	**Obesity**
**History Preeclampsia**	75	87.50	50.00	66.67	93.33	83.33
**Chronic Hypertension**		71.43	100	77.78	89.47	77.78
**Pregestational Diabetes**			45	45.45	83.33	57.14
**Advanced Maternal Age**				31.78	48.39	27.12
**Black race **					32.93	32.14
**Obesity**						23.74

## Discussion/Conclusions

In this study we aimed to examine the associations between preeclampsia risk factors and aspirin prescribing practices among providers in a network of Federally Qualified Health Centers. Identifying missed opportunities in aspirin prescribing is an important part of efforts to improve health outcomes in populations at higher risk for maternal morbidity and mortality. Our results demonstrate that of the 249 total patients who were considered at risk for preeclampsia and therefore eligible for aspirin prophylaxis under ACOG and USPSTF guidelines, only 39% received a prescription. This low rate of appropriately prescribed aspirin was mainly due to gaps in providing prophylaxis to eligible patients with multiple moderate-risk factors without comorbid high-risk factors.

Although 57.89% of patients with any high-risk factor were appropriately prescribed aspirin, only 27.27% of patients with two or more moderate-risk factors without high-risk factors were prescribed aspirin, highlighting the need for improvement in clinician recognition of preeclampsia moderate-risk factors. History of preeclampsia and chronic hypertension were the risk factors for which clinicians most frequently prescribed aspirin, suggesting that they may recognize these high-risk factors more often than any other preeclampsia risk factors. However, despite this greater recognition, aspirin was prescribed a maximum of 78.79% of the time for patients with chronic hypertension, leaving a significant proportion of high-risk patients without recommended prophylaxis. Among moderate-risk factors, only patients with AMA, Black race, or nulliparity were significantly more likely in adjusted models to be prescribed aspirin.

Among limited studies assessing strategies for improving aspirin prescribing practices, implementation of standardized preeclampsia risk screening in the electronic health record has been associated with the most significant improvement [16]. These tools may help to increase identification of patients with moderate-risk factors for preeclampsia, for whom provision of low-dose aspirin is recommended per professional guidelines.

Our study also demonstrates that certain sociodemographic factors such as race/ethnicity play a role in determining who receives aspirin for preeclampsia prevention. Among moderate- risk factors, Black race was associated with a disproportionately higher number of aspirin prescriptions compared to all other moderate-risk factors, suggesting a potential bias among healthcare providers. While it is important to recognize the significantly greater pregnancy-related morbidity and mortality among Black birthing individuals, attributing this risk solely to race can overlook health inequities and oversimplify the complex influence of structural racism. Moreover, the available evidence on aspirin prophylaxis for preeclampsia prevention in Black birthing individuals is limited, and results of systematic reviews, when treatment effects are stratified by race, have not demonstrated statistically significant differences [12].

These results highlight the need for clinician-facing education in community health centers providing prenatal care to high-risk populations. Studies reporting similarly low rates of aspirin prescribing in high-risk populations suggest that despite the established benefits of aspirin and clear recommendations from professional societies, there are still many missed opportunities for aspirin prophylaxis to prevent preeclampsia [17]. Interventions need to focus on identifying patients who qualify for aspirin prophylaxis on the basis of having multiple moderate -risk factors without comorbid high-risk factors. Among the moderate-risk factors, providers should not prescribe aspirin based on a single moderate-risk factor alone, as this may indicate bias or a heightened awareness of the association between certain risk factors and ignore others. Future interventions may consider training or clinical decision support tools in the electronic health record aimed at helping providers more quickly identify patients with these multiple moderate-risk factors.

Limitations of our study included availability and completeness of delivery data in the electronic medical record. In addition, due to the retrospective nature of this study, some risk factors were not reliably documented and available in the prenatal record: family history of preeclampsia in a first degree relative, low socioeconomic status, history of low birthweight or small for gestational age infant, previous adverse pregnancy outcome, or interpregnancy interval greater than ten years. We were also unable to assess patient adherence to aspirin therapy, which has implications for future efforts to assess preeclampsia outcomes.

These findings have important implications for clinical practice in similar community health settings. Under existing ACOG guidelines “low income” is considered a moderate-risk factor for preeclampsia. Our practice does not yet have a way to formally define, screen for, and include this risk factor in aspirin-prescribing decisions, but insurance status may be considered a proxy. 80% of our Federally Qualified Health Center patients included in this study carry Medicaid insurance, aimed at providing health coverage for low-income individuals. Future research efforts might consider implementing a universal opt-out strategy to increase aspirin prescription rates in similar clinical settings. This is supported by the latest ACOG guidelines, which state that universal implementation may be medically reasonable in practices where the majority of patients may be at high or moderate risk for preeclampsia. Improving aspirin prescribing rates for preeclampsia prevention has the potential to significantly reduce the burden of this condition on both individuals and their newborns. In addition, addressing disparities in aspirin prescribing based on patient characteristics may help to reduce persistent disparities in maternal health outcomes.

## Data Availability

A fully de-identified data set is available from the corresponding author on reasonable request.

## Abbreviations

HDP: Hypertensive disorders of pregnancy
MM: Maternal mortality
SMM: Severe maternal morbidity
PEC: Global rates of preeclampsia
FQHC: Federally qualified health center
USPSTF: United States Preventive Services Task Force
ACOG: American College of Obstetricians and Gynecologists
AMA: Advanced maternal age
ASA: Aspirin
